# COVID-19 prevention and control effect of non-pharmaceutical interventions—fuzzy-sets qualitative comparative analysis based on 69 countries in the world

**DOI:** 10.3389/fpubh.2024.1419109

**Published:** 2024-07-26

**Authors:** Chunxiao Yang, Qiusha Li, Zixuan Zhao, Zhongming Chen, Hongwei Guo, Dongmei Huang, Wenqiang Yin

**Affiliations:** ^1^School of Public Health, Shandong Second Medical University, Weifang, Shandong, China; ^2^School of Management, Shandong Second Medical University, Weifang, Shandong, China

**Keywords:** COVID-19, non-pharmaceutical interventions, fuzzy-sets qualitative comparative analysis, configurations, public health

## Abstract

**Introduction:**

Coronavirus disease 2019 occurred unexpectedly in late December 2019, it was difficult to immediately develop an effective vaccine or propose targeted medical interventions in the early stages of the outbreak. At this point, non-pharmaceutical interventions (NPIs) are essential components of the public health response to COVID-19. How to combine different NPIs in the early stages of an outbreak to control the spread of epidemics and ensure that the policy combination does not incur high socio-economic costs became the focus of this study.

**Methods:**

We mainly used the fuzzy set qualitative comparative analysis to assess the impact of different combinations of NPIs on the effectiveness of control in the COVID-19 pandemic early stage, using open datasets containing case numbers, country populations and policy responses.

**Results:**

We showed that the configuration of high morbidity results includes one, which is the combination of non-strict face covering, social isolation and travel restrictions. The configuration of non-high morbidity results includes three, one is strict mask wearing measures, which alone constitute sufficient conditions for interpreting the results; the second is strict testing and contact tracing, social isolation; the third is strict testing and contact tracing, travel restriction. The results of the robustness test showed that the number, components and consistency of the configurations have not changed after changing the minimum case frequency, which proved that the analysis results are reliable.

**Conclusion:**

In the early stages of the epidemic, the causes of high morbidity are not symmetrical with the causes of non-high morbidity. Strict face covering is the most basic measure required to prevent and control epidemics, and the combination of non-strict face covering and containment is the most important factor leading to poor prevention and control, and the combination of strict containment and proactive pursuit is the way to achieve superior prevention and control, timely and proactive containment strategies have better prevention and control, and should mobilize the public to cooperate.

## 1 Introduction

In late December 2019, COVID-19 occurred unexpectedly. On 11 March 2020, the World Health Organization (WHO) declared coronavirus disease 2019 (COVID-19) a pandemic (1). By the end of March 2020, and based on data from the Our World in Data-Coronavirus (COVID-19) Cases, more than 200 countries worldwide have reported cases (2). During an outbreak, non-pharmaceutical interventions (NPIs) and pharmaceutical interventions can play an important synergistic role in the prevention and control of the epidemic. However, in the early stages of an epidemic, effective pharmaceutical interventions are not expected to be available (3), NPIs are essential components of the public health response to COVID-19 (4–6).

Non-Pharmaceutical Interventions (NPIs) are public health measures to reduce transmission by reducing exposure in the general population (7). The primary objectives of NPIs are to reduce the likelihood of transmission, thereby minimizing the magnitude and delaying the arrival of peak outbreaks. This will buy time for health systems to prepare, and increase the potential for the development, approval and use of vaccines or drugs (8, 9). Current evidence suggests that single interventions have a more limited effect in the early stages of an epidemic and that multiple interventions are required to influence disease transmission (7, 10). However, the more interventions that are implemented, the higher the social and economic costs. Therefore, we focus on how to combine different interventions to control the spread of the epidemic and ensure that the multiple interventions do not result in high socioeconomic costs.

As there are many NPIs that affect the effectiveness of epidemic prevention and control, there is no independence between different NPIs; the impact of specific NPIs cannot be evaluated in isolation (5). Previous studies have used traditional quantitative methods to explore the net effect of individual NPIs, but lack studies on the combinations of NPIs that affect control effectiveness. Qualitative comparative analysis (QCA) is a more appropriate approach for such a complex causal research situation. QCA is a method that lies between quantitative and qualitative research. Unlike causal inference based on correlations between variables, QCA is based on the aggregation of the set of conditions and the set of outcomes. It focuses on the adequate and necessary conditions for an outcome to occur, and pays more attention to how multiple different antecedent elements influence the final outcome in the form of “configuration.” Currently, crisp-sets qualitative comparative analysis (csQCA), fuzzy-sets qualitative comparative analysis (fsQCA), and multi-value qualitative comparative analysis (mvQCA) are the more widely used QCA methods. csQCA and mvQCA are only suitable for dealing with kind problems, i.e., the cases can only be assigned to a certain category of categorical variables. However, fsQCA can not only deal with the kind problem, but also deal with the degree of change and partial membership. It introduces the concept of affiliation (the degree of certain attributes in the case), and distinguishes the original variables into the set of partial membership or partial non-membership, which can effectively avoid the problem of information loss in the process of data transformation. Since fsQCA has greater advantages, where possible, the researcher suggests that fsQCA should be used for analysis (11). In this study, the effectiveness of epidemic prevention and control were difficult to be classified simply by categories. Therefore, we used fsQCA to explore the influence of different combinations of NPIs on the epidemic prevention and control effect in the early stage so as to provide theoretical and practical insights for early response to emerging infectious disease outbreaks.

## 2 Materials and methods

### 2.1 Study design

This study focused on the window period when the cumulative number of cases in each country exceeds 100. By this time, the outbreak had begun to take shape and control measures were critical (12). Therefore, we used the point at which countries had accumulated more than 100 confirmed cases as the starting point for data collection. According to the experience of major countries, the spread of the epidemic can be gradually controlled in about 6 weeks if combined with effective epidemic prevention measures (13). Therefore, the cut-off point for data collection was the first day after the cumulative number of confirmed cases in each country exceeded 100 for 6 weeks. We collected data of time starting points and cut-off points of each country, built a case base and conducted configuration analysis.

### 2.2 Materials

In this study, the data of the outcome variable were obtained from the Our World in Data, a thematic database of COVID-19 statistics, which was compiled from information provided by countries’ official websites (government, ministry of health, or CDC, etc.) (2). We mainly used number of cases when the cumulative cases exceeded 100, the cumulative number of confirmed cases after 6 weeks, the population of each country.

The data of the condition variables were obtained from the Oxford COVID-19 Government Response Tracker (OxCGRT) (14), a dataset that provides systematic information on government responses to COVID-19 collected from public sources. We collected policy stringency scores for each country’s four condition variables: face covering, testing and contact tracing, social isolation, and travel restrictions.

### 2.3 Methods

We mainly used the fuzzy-set qualitative comparative analysis (fsQCA) to assess the impact of different combinations of NPIs on the effectiveness of control in the COVID-19 pandemic early stage.

First, we identified conditional variables using the literature induction method. Literature induction method is one of the main methods for determining condition variables in QCA research, which refers to the induction of important conditions from existing related literature (15). Based on this method, we used face covering, social isolation, travel restriction, testing, and contact tracing as conditional variables, and 6-week morbidity as outcome variables. Second, we constructed a final case base of 69 countries on the methodological principles of fsQCA. Third, we calibrated the data using the direct calibration method. Calibration involves calibrating the data by converting conventional variables to fuzzy variables using the fsQCA analysis software. In the case of morbidity, for example, the values of the variables before calibration only reflect the level of morbidity in each country, while after calibration it is possible to define which countries are affiliated with high morbidity and which countries are affiliated with low morbidity. Calibration is divided into two methods, direct and indirect, with the indirect method requiring a qualitative assessment of pooled affiliation. In order to avoid possible errors or controversies due to the subjective use of the indirect method, this study used the direct calibration method for calibration. That is, the raw absolute values were converted to fuzzy affiliation scores based on the three anchor points for constructing a fuzzy set: full membership, full non-membership, and crossover point. Finally, we analyzed the necessity, the adequacy of the conditional configuration and conducted a robustness test.

### 2.4 Variable selection

#### 2.4.1 Conditional variables

Previous studies usually focus on NPIs that directly affect the probability of exposure, such as masking, social isolation, case isolation, testing and contact tracing, travel restrictions, and stay-at-home restrictions (4, 16, 17). Therefore, this study also analyzed NPIs that directly affect the probability of exposure.

In this study, based on the literature induction method (15), we systematically summarized the NPIs that control the transmission rate of COVID-19 mainly contain three categories: (1) Basic protective measures that the government requires individuals to comply with. The role of face covering in avoiding contact between infected and susceptible individuals is representative, and it has been required and implemented as a clear policy, so it is selected as one of the conditional variables (18, 19); (2) Closure measures to restrict the movement and gathering of people. NPIs that restrict the movement of people are mainly travel restrictions; NPIs that restrict the gathering of people, i.e., social isolation measures that include closing schools and workplace, canceling public events and restrictions on gatherings, which are designed to avoid the occurrence of clusters of cases (19, 20). In existing studies. Stay-at-home restrictions is also included as a closure measure, but given that it is a combination of both gathering and movement measures, there may be cross-over effects between the measures, therefore stay-at-home restrictions is not included in the analysis; (3) The chase measure to identify infected and potentially infected persons. This is a proactive case detection measure based on contact intervention that can identify and isolate infected persons as soon as possible to prevent the spread of the virus. Based on existing studies and the primary purpose of implementing NPIs, the four main NPIs: face covering measures, testing and contact tracing, social isolation, and travel restrictions were included in the study.

Therefore, in this study, face covering, travel restrictions, social isolation, testing and contact tracing were used as condition variables, where the values of face covering was obtained by using initial values from the Oxford COVID-19 Government Response Tracker (OxCGRT); travel restrictions was obtained by calculating the mean using domestic travel restrictions, with values ranging from [0, 4]; close public transport; social isolation was obtained by calculating the mean using restrictions on gatherings, cancelation of public events, school closures, and workplace closures with values ranging from [0, 3]; testing and contact tracing was obtained by calculating the mean using testing policy and contact tracing, with values ranging from [0, 2.5]. The values of conditional variables represents the stringency of the NPIs, with the lowest value of 0 indicating no restriction, and the higher the score, the more stringent the intervention (21).

#### 2.4.2 Outcome variables

Epidemiologically, it is believed that through the comparison of morbidity, the epidemic characteristics of the disease can be understood and the effect of prevention and control measures can be evaluated (22). Therefore, the 6-week morbidity per 100,000 population was used as the outcome variable. The specific formula was: 6-week morbidity = (number of new cases at 6 weeks/population) × 1/100,000. The 6-week number of new cases is the difference between the number of confirmed cases diagnosed at the time point of more than 100 cases in each country and the number of confirmed cases diagnosed at the time point 6 weeks later.

### 2.5 Case selection

Case selection of QCA is an experimental and iterative process, so random sampling cannot be simply adopted, sufficient homogeneity of the case population and maximum heterogeneity within the case population should be considered (23). Based on this principle, we mainly conducted two screening.

#### 2.5.1 Initial screening

Based on the principle of sufficient homogeneity, a globally representative case base was preliminarily established according to the following four steps: (1) Since sovereign states have the sovereign right to act and rule within their borders, the data of 197 sovereign states in “Our World in Data” is collected; (2) Countries with populations over 5 million are selected to ensure that the pandemic containment experience applicable to large countries is extracted; (3) To ensure that the data of each condition variable are complete when 100 cases are diagnosed in the selected country; (4) Only countries that have passed at least 6 weeks since the 100th case was confirmed are included, these countries already have a certain number of cases and have had enough time to respond so that low prevalence can be the result of effective containment (24). Initial screening to establish the initial cases containing 119 countries, basic information on the initial case base of 119 cases is shown in Tables 1, 2.

**Table 1:** Initial case base of 119 cases.

Countries	Population	More than 100 cases in time	Time after 6 weeks	Face covering	Testing and	Social isolation	Travel restrictions	6-week morbidity
					Contact tracing			
Afghanistan	39,835,428	2020/3/28	2020/5/10	0	1	2	3	10.79
Algeria	44,616,626	2020/3/21	2020/5/3	0	0	1.25	0	9.72
Angola	33,933,611	2020/6/10	2020/7/23	3	1	2.5	3	2.17
Argentina	45,605,823	2020/3/20	2020/5/2	0	1.5	3	2	9.98
Australia	25,788,217	2020/3/10	2020/4/22	0	1.5	0	0	25.38
Austria	9,043,072	2020/3/9	2020/4/21	0	1	0	0	163.02
Azerbaijan	10,223,344	2020/3/26	2020/5/8	0	2	2.75	2	21.1
Bangladesh	166,303,494	2020/4/6	2020/5/19	0	1.5	3	4	15.03
Belarus	9,442,867	2020/3/30	2020/5/12	1	0	0	0	261.8
Belgium	11,632,334	2020/3/5	2020/4/17	0	1.5	0	0	310.24
Benin	12,451,031	2020/5/7	2020/6/19	3	2	2.75	4	4.1
Bolivia	11,832,936	2020/3/31	2020/5/13	0	0	3	4	25.7
Brazil	213,993,441	2020/3/13	2020/4/25	1	0.5	1.5	0	27.65
Bulgaria	6,896,655	2020/3/20	2020/5/2	0	0.5	2.5	2	21.27
Burkina Faso	21,497,097	2020/3/24	2020/5/6	0	1.5	2	4	2.86
Burundi	12,255,429	2020/6/16	2020/7/29	0	1	0	0	2.31
Cambodia	16,946,446	2020/3/29	2020/5/11	0	1	2.75	0	0.11
Cameroon	27,224,262	2020/3/29	2020/5/11	0	0.5	2.5	2	9.37
Canada	38,067,913	2020/3/13	2020/4/25	0	1.5	0.5	0	121.25
Chad	16,914,985	2020/5/2	2020/6/14	0	1	2.75	4	4.33
Chile	19,212,362	2020/3/15	2020/4/27	0	0	0.75	0	80.11
China	1,444,216,102	2020/1/19	2020/3/2	1	1.5	0	2	5.53
Colombia	51,265,841	2020/3/18	2020/4/30	0	1	2.25	0	12.49
Congo	5,657,017	2020/4/15	2020/5/28	0	1	2.75	4	8.03
Costa Rica	5,139,053	2020/3/21	2020/5/3	0	1.5	1.75	4	12.1
Cote d’Ivoire	27,053,629	2020/3/27	2020/5/9	0	1.5	2.5	3	5.79
Cuba	11,317,498	2020/3/28	2020/5/10	0	1.5	1.25	4	14.55
Czechia	10,724,553	2020/3/13	2020/4/25	0	1.5	2.5	0	67.24
Democratic Republic of Congo	92,377,986	2020/4/1	2020/5/14	1	1	2.5	4	1.23
Denmark	5,813,302	2020/3/10	2020/4/22	0	0.5	0.25	1	134.97
Dominican Republic	10,953,714	2020/3/21	2020/5/3	0	0.5	3	4	71.59
Ecuador	17,888,474	2020/3/18	2020/4/30	2	0.5	2.75	4	138.77
Egypt	104,258,327	2020/3/14	2020/4/26	0	1.5	0	0	4.24
El Salvador	6,518,500	2020/4/9	2020/5/22	3	1	2.5	3	24.88
Ethiopia	117,876,226	2020/4/18	2020/5/31	3	1	2.75	4	0.91
Finland	5,548,361	2020/3/13	2020/4/25	0	1	1	0	74.42
France	67,564,251	2020/3/1	2020/4/13	1	1.5	1.5	0	165.48
Germany	83,900,471	2020/3/1	2020/4/13	0	1.5	0.75	0	154.88
Ghana	31,732,128	2020/3/26	2020/5/8	0	1.5	2	0	12.23
Greece	10,370,747	2020/3/13	2020/4/25	0	0	1.75	0	22.33
Guatemala	18,249,868	2020/4/10	2020/5/23	3	1	3	4	16.04
Guinea	13,497,237	2020/4/4	2020/5/17	0	1.5	2.5	3	18.87
Haiti	11,541,683	2020/5/6	2020/6/18	2	0.5	2.75	4	38.53
Honduras	10,062,994	2020/3/29	2020/5/11	0	1	3	4	19.78
Hungary	9,634,162	2020/3/21	2020/5/3	0	1.5	2	2	30.05
India	1,393,409,033	2020/3/14	2020/4/26	0	1.5	1.25	0	1.99
Indonesia	276,361,788	2020/3/15	2020/4/27	0	1	1.5	0	3.25
Iran	85,028,760	2020/2/26	2020/4/9	0	0	0.75	0	77.72
Iraq	41,179,351	2020/3/13	2020/4/25	0	1	2.25	4	4.04
Israel	9,291,000	2020/3/14	2020/4/26	0	1	2	0	158.83
Italy	60,367,471	2020/2/23	2020/4/6	0	1.5	3	1	219.31
Japan	126,050,796	2020/2/22	2020/4/5	0	1	0.25	0	2.97
Jordan	10,269,022	2020/3/22	2020/5/4	0	1.5	3	4	3.44
Kazakhstan	18,994,958	2020/3/26	2020/5/8	1	1.5	2.25	4	24.86
Kenya	54,985,702	2020/4/2	2020/5/15	0	1	2.75	3	1.22
Kyrgyzstan	6,628,347	2020/3/31	2020/5/13	0	1	3	4	14.14
Lebanon	6,769,151	2020/3/15	2020/4/27	2	1	1.25	0	8.86
Liberia	5,180,208	2020/4/21	2020/6/3	0	1	3	3	4.19
Libya	6,958,538	2020/5/28	2020/7/10	0	1	3	4	17.78
Madagascar	28,427,333	2020/4/11	2020/5/24	0	1	2.75	4	1.5
Malawi	19,647,681	2020/5/25	2020/7/7	1	1.5	2.25	0	8.74
Malaysia	32,776,195	2020/3/9	2020/4/21	2	1.5	0.25	0	16.37
Mali	20,855,724	2020/4/12	2020/5/25	0	0	2.5	2	4.57
Mexico	130,262,220	2020/3/18	2020/4/30	0	0.5	0	0	14.67
Morocco	37,344,787	2020/3/22	2020/5/4	0	1	2.75	4	13.22
Mozambique	32,163,045	2020/5/11	2020/6/23	3	1.5	2	1	2.03
Myanmar	54,806,014	2020/4/19	2020/6/1	0	1.5	3	4	0.21
Nepal	29,674,920	2020/5/7	2020/6/19	0	1.5	2.75	4	27.54
Netherlands	17,173,094	2020/3/8	2020/4/20	0	1.5	0	0	194.04
New Zealand	5,122,600	2020/3/22	2020/5/4	0	1.5	1.75	1	27.02
Nicaragua	6,702,379	2020/5/19	2020/7/1	0	1	0.5	0	33.79
Niger	25,130,810	2020/4/3	2020/5/16	0	1	2	2	3.06
Nigeria	211,400,704	2020/3/29	2020/5/11	0	1.5	3	4	2.14
Norway	5,465,629	2020/3/7	2020/4/19	0	0.5	0	0	126.81
Oman	5,223,376	2020/3/26	2020/5/8	1	2	1	0	57.49
Pakistan	225,199,929	2020/3/15	2020/4/27	0	1	2.25	4	6.43
Palestine	5,222,756	2020/3/29	2020/5/11	0	1	2.5	3	5.09
Papua New Guinea	9,119,005	2020/8/2	2020/9/14	0	1.5	1.5	4	4.4
Paraguay	7,219,641	2020/4/5	2020/5/18	3	2	3	3	9.35
Peru	33,359,415	2020/3/17	2020/4/29	3	1	3	3	101.36
Philippines	111,046,910	2020/3/14	2020/4/26	0	2	2	4	6.73
Poland	37,797,000	2020/3/14	2020/4/26	0	0	1.75	1	30.46
Portugal	10,167,923	2020/3/13	2020/4/25	0	1	2.25	0	228.96
Romania	19,127,772	2020/3/14	2020/4/26	0	0	2.25	0	57.05
Russia	145,912,022	2020/3/17	2020/4/29	0	1.5	1.5	2	68.04
Rwanda	13,276,517	2020/4/4	2020/5/17	0	2.5	2.5	0	1.43
Saudi Arabia	35,340,680	2020/3/14	2020/4/26	0	2	2	4	49.29
Senegal	17,196,308	2020/3/26	2020/5/8	0	1.5	1.75	0	8.41
Serbia	6,871,547	2020/3/19	2020/5/1	0	1.5	2.5	4	129.61
Sierra Leone	8,141,343	2020/4/28	2020/6/10	0	2	1.75	0	11.77
Singapore	5,453,600	2020/3/1	2020/4/13	4	2	1	2	51.56
Slovakia	5,460,726	2020/3/18	2020/4/30	0	2	1.25	0	23.81
Somalia	16,359,500	2020/4/17	2020/5/30	0	0.5	1	0	11
South Africa	60,041,996	2020/3/18	2020/4/30	1	1.5	2	3	9.21
South Korea	51,305,184	2020/2/20	2020/4/3	1	2.5	1.75	1	19.41
South Sudan	11,381,377	2020/5/8	2020/6/20	0	2	1.25	0	15.48
Spain	46,745,211	2020/3/2	2020/4/14	2	1	1.5	2	368.85
Sri Lanka	21,497,306	2020/3/24	2020/5/6	0	1.5	1.5	0	3.23
Sudan	44,909,351	2020/4/21	2020/6/3	0	1.5	2.75	1	11.59
Sweden	10,160,159	2020/3/6	2020/4/18	0	1.5	1.25	2	139.06
Switzerland	8,715,494	2020/3/4	2020/4/16	0	1.5	0.75	0	305.69
Syria	18,275,704	2020/5/25	2020/7/7	0	1	1.25	0	1.46
Tajikistan	9,749,625	2020/5/3	2020/6/15	2	1	0.5	0	51.5
Tanzania	61,498,438	2020/4/17	2020/5/30	2	0.5	2	0	0.59
Thailand	69,950,844	2020/3/15	2020/4/27	3	1	1.5	3	4.03
Togo	8,478,242	2020/4/29	2020/6/11	0	0.5	1.25	0	4.89
Tunisia	11,935,764	2020/3/24	2020/5/6	0	1	3	3	7.63
Turkey	85,042,736	2020/3/19	2020/5/1	0	1.5	1.75	2	143.69
Uganda	47,123,533	2020/5/6	2020/6/18	0	1	1.75	1	1.36
Ukraine	43,466,822	2020/3/25	2020/5/7	3	1	2.75	4	31.16
United Arab Emirates	9,991,083	2020/3/18	2020/4/30	0	1.5	1.5	2	123.79
United Kingdom	68,207,114	2020/3/4	2020/4/16	0	1.5	0.25	0	165.03
United States	3.33E+08	2020/3/4	2020/4/16	0	1.5	0.25	0	205.01
Uzbekistan	33,935,765	2020/3/28	2020/5/10	0	1.5	1.5	0	6.82
Venezuela	28,704,947	2020/3/26	2020/5/8	3	0.5	2.75	2	0.98
Vietnam	98,168,829	2020/3/22	2020/5/4	2	2	1.75	2	0.16
Yemen	30,490,639	2020/5/15	2020/6/27	0	0	1.75	2	3.27
Zambia	18,920,657	2020/4/30	2020/6/12	3	1.5	1.5	0	6.42
Zimbabwe	15,092,171	2020/5/27	2020/7/9	0	1	2.75	3	4.99

**Table 2:** Basic information of the initial case base constructed from 119 samples.

Variable category	Variable name	Minimum	5% quartile	Median	95% quartile	Maximum
Result variable	Morbidity	0.11	1.20	14.14	206.44	368.85
Conditional variables	Face covering	0	0	0	3	4
	Testing and contact tracing	0	0	1	2	2.5
	Social isolation	0	0	2	3	3
	Travel restrictions	0	0	2	4	4

#### 2.5.2 Secondary screening

Based on the principle of maximum heterogeneity, the final case-base for inclusion in the fsQCA analysis was established according to the following criteria: (1) Ensure that included cases have sufficient “positive” outcomes with morbidity below the median and “negative” outcomes with morbidity above the median; (2) Ensure that the representativeness of the selected countries, at the same time, in order to avoid the loss of the value of conditional variables. The value of each condition variable is guaranteed to correspond to a certain number of cases, so as to ensure that there are corresponding cases in each combination of condition variables; (3) Excluding countries with large volatility in the data of confirmed cases; and (4) According to the characteristics and effects of prevention and control measures of major countries and the degree of grasping case information, 69 cases were selected to form the final case database (Tables 3, 4).

**Table 3:** Final case base of 69 cases.

Countries	Face covering	Testing and contact tracing	Social isolation	Travel restrictions	6-week morbidity
Angola	3	1.00	2.50	3	2.17
Argentina	0	1.50	3.00	2	9.98
Australia	0	1.50	0.00	0	25.38
Austria	0	1.00	0.00	0	163.02
Azerbaijan	0	2.00	2.75	2	21.1
Bangladesh	0	1.50	3.00	4	15.03
Belarus	1	0.00	0.00	0	261.8
Benin	3	2.00	2.75	4	4.1
Bolivia	0	0.00	3.00	4	25.7
Brazil	1	0.50	1.50	0	27.65
Bulgaria	0	0.50	2.50	2	21.27
Burkina Faso	0	1.50	2.00	4	2.86
Canada	0	1.50	0.5	0	121.25
Chile	0	0.00	0.75	0	80.11
China	1	1.50	0	2	5.53
Costa Rica	0	1.50	1.75	4	12.1
Cote d’Ivoire	0	1.50	2.5	3	5.79
Cuba	0	1.50	1.25	4	14.55
Czechia	0	1.50	2.50	0	67.24
Denmark	0	0.50	0.25	1	134.97
Dominican Republic	0	0.50	3.00	4	71.59
Ethiopia	3	1.00	2.75	4	0.91
Finland	0	1.00	1.00	0	74.42
France	1	1.50	1.50	0	165.48
Germany	0	1.50	0.75	0	154.88
Ghana	0	1.50	2.00	0	12.23
Guatemala	3	1.00	3.00	4	16.04
Guinea	0	1.50	2.50	3	18.87
Honduras	0	1.00	3.00.	4	19.78
Hungary	0	1.50	2.00	2	30.05
Iran	0	0.00	0.75	0	77.72
Italy	0	1.50	3.00	1	219.31
Japan	0	1.00	0.25	0	2.97
Jordan	0	1.50	3.00	4	3.44
Kyrgyzstan	0	1.00	3.00	4	14.14
Lebanon	2	1.00	1.25	0	8.86
Libya	0	1.00	3.00	4	17.78
Malawi	1	1.50	2.25	0	8.74
Mexico	0	0.50	0.00	0	14.67
Mozambique	3	1.50	2.00	1	2.03
Myanmar	0	1.50	3.00	4	0.21
Nepal	0	1.50	2.75	4	27.54
Netherlands	0	1.50	0.00	0	194.04
New Zealand	0	1.50	1.75	1	27.02
Nicaragua	0	1.00	0.50	0	33.79
Nigeria	0	1.50	3.00	4	2.14
Norway	0	0.50	0.00	0	126.81
Paraguay	3	2.00	3.00	3	9.35
Philippines	0	2.00	2.00	4	6.73
Rwanda	0	2.50	2.50	0	1.43
Saudi Arabia	0	2.00	2.00	4	49.29
Senegal	0	1.50	1.75	0	8.41
Serbia	0	1.50	2.50	4	129.61
Sierra Leone	0	2.00	1.75	0	11.77
Singapore	4	2.00	1.00	2	51.56
South Africa	1	1.50	2.00	3	9.21
South Korea	1	2.5	1.75	1	19.41
Spain	2	1.00	1.50	2	368.85
Sudan	0	1.50	2.75	1	11.59
Sweden	0	1.50	1.25	2	139.06
Switzerland	0	1.50	0.75	0	305.69
Tanzania	2	0.50	2.00	0	0.59
Thailand	3	1.00	1.50	3	4.03
Turkey	0	1.50	1.75	2	143.69
United Kingdom	0	1.50	0.25	0	165.03
United States	0	1.50	0.25	0	205.01
Venezuela	3	0.50	2.75	2	0.98
Vietnam	2	2.00	1.75	2	0.16
Zambia	3	1.50	1.50	0	6.42

**Table 4:** Basic information of the final case base constructed from 69 samples.

Variable category	Variable Name	Minimum	5% quartile	Median	95% quartile	Maximum
Result variable	Morbidity	0.16	0.94	18.87	213.59	368.85
Conditional variables	Face covering	0	0	0	3	4
	Testing and contact tracing	0	0.20	1.50	2	2.5
	Social isolation	0	0	2	3	3
	Travel restrictions	0	0	2	4	4

The selected cases are all sovereign states with a certain population size, which basically meets the homogeneity requirement of case selection. The values of condition and outcome variables in case countries have great heterogeneity, which meets the diversified requirements of case selection. Geographically, the 69 cases cover countries from six continents, including 17 countries in Asia, 18 countries in Africa, 17 countries in Europe, six countries in South America, nine countries in North America, and two countries in Oceania.

### 2.6 Variable calibration

Calibration is the process of converting conventional variables into fuzzy variables. In terms of setting the anchor points, Ragin recommends that existing standards should be adopted if there are specific descriptions of variables in existing studies (23). The value of the face covering was determined by two points: (1) the initial value of the “Our World in Data” variable; (2) In the early stage of the epidemic, more countries adopted relatively relaxed measures to wear masks. Therefore, the threshold for full membership of the face covering is 2, when the mask must be worn outside the house in the presence of others in certain shared/public Spaces or in cases where social evacuation is not possible; The value of the crossover point is 1, when the face covering measures introduced are not mandatory, but only recommended; the threshold of full non-membership is 0, which means no measures have been put in place to wear masks. For other variables, since there is no existing information as criteria for threshold setting, we adopted the experience of Andrews and other scholars’ studies, setting them based on statistical values, using the 5% quantile of the original data as a fully non-membership threshold, using the 95% quantile as a fully membership threshold, and using the median as a crossover point (25). In this case, considering the scientific and representative nature of the 6-week morbidity set division between high and non-high, its threshold was set according to the quartile of the initial database containing 119 cases. The specific thresholds set are shown in Table 5.

**Table 5:** Calibration taking criteria for outcome and condition variables.

Variable category	Target variables	Calibration method	Calibration		
			Full membership	Crossover point	Full non-membership
Result variables	High morbidity	Direct calibration	206.44	14.14	1.20
	Non-high morbidity	Direct calibration	1.20	14.14	206.44
Conditional variables	Strict face covering	Direct calibration	2.00	1.00	0.00
	Strict testing and contact tracing	Direct calibration	2.00	1.50	0.20
	Strict social isolation	Direct calibration	3.00	2.00	0.00
	Strict travel restrictions	Direct calibration	4.00	2.00	0.00

## 3 Results

### 3.1 Individual conditions: test of necessity

The necessity analysis of individual condition tests whether the outcome is a subset of a certain set of conditions. Necessity conditions are measured by consistency, if the consistency of a condition variable is above 0.9; it is the necessary antecedent condition of the result variable (26). The results of the test shows that the level of consistency of all conditions is not higher than 0.9, there is no absolutely necessary condition affecting the morbidity of each country among the four condition variables (Table 6).

**Table 6:** Individual conditions: test of necessity.

Condition variables	High morbidity		Non-high morbidity	
	Consistency	Coverage	Consistency	Coverage
Face covering (FC)	0.245	0.414	0.438	0.793
~ FM	0.878	0.593	0.676	0.490
Testing and contact tracing (TCT)	0.562	0.612	0.627	0.731
~ TCT	0.753	0.653	0.667	0.620
Social isolation (SI)	0.538	0.510	0.726	0.738
~SI	0.724	0.711	0.518	0.545
Travel restrictions (TR)	0.445	0.480	0.626	0.723
~TR	0.743	0.650	0.549	0.514

FC, TCT, SI, and TR are four measures abbreviated, and “~” means “non,” which is the inverse set of this variable and represents the non-strict counterpart measure; the coverage itself has no reference meaning, but judges the strength of empirical interpretation of the subset relation after it is confirmed.

### 3.2 Adequate configuration analysis

Adequate configuration analysis of conditional configuration is the core of the QCA, which mainly analyzes the sufficiency of configuration formed by different antecedent conditions on the results (15). First, we set case frequency as 1, set the consistency threshold set as 0.75 by referring to Ragin’s suggestion. Second, before the analysis, Proportional Reduction in Inconsistency (PRI) was combined to further filter the truth table. According to the suggestions of leading scholars, we took 0.5 as the PRI threshold (27).

By using fsQCA3.0 software to carry out Boolean minimization operation on the truth table, we obtained complex solutions, parsimonious solutions and intermediate solutions. More and more scholars have found in empirical research that both complex solutions and intermediate solutions fail to pass basic correctness tests and are prone to causal fallacies. Researchers who use complex solutions or intermediate solutions in empirical analysis always run the risk of being far from the truth rather than closer to it (28–30). In this study, the economic and social costs of introducing measures should be taken into account, so we are committed to find the necessary combinations to promote the effectiveness of prevention, focusing on realism and simplicity. Parsimonious solutions with more simplicity and less risk of error are more suitable for analysis. After testing, the consistency of the parsimonious solutions met the threshold above 0.75, proving that each grouping has been a sufficient condition for the results, so we choose to report the parsimonious solutions, interpret it according to the standardized reporting symbols (31, 32).

For the four configurations presented in Table 7, the consistency level of both single solution (configuration) and overall solution is higher than the minim acceptable standard of 0.75. There were one configuration with high morbidity and three configurations with non-high morbidity.

**Table 7:** Configuration analysis.

Condition variables	High morbidity	Non-high morbidity		
	Configuration	Configuration 1	Configuration 2	Configuration 3
Face covering	⊗	•		
Testing and contact tracing			•	•
Social isolation	⊗		•	
Travel restrictions	⊗			•
Consistency	0.826	0.793	0.862	0.854
Original coverage	0.581	0.438	0.525	0.435
Unique coverage	0.581	0.182	0.086	0.020
Consistency of the overall solution	0.826	0.787		
Overall solution coverage	0.581	0.748		

⊗ indicates that the condition variable does not appear, • indicates that the condition variable appears, and a space indicates that the condition variable may or may not appear in the configuration.

#### 3.2.1 Configuration outcomes of high morbidity

The combination of non-high testing and contact tracing, social isolation and travel restrictions constituted a high morbidity outcome. The consistency of the configuration was 0.826 and the original coverage was 0.581, suggesting that the path could explain 58.1% of cases. The high morbidity was the result of the combination of three factors: non-strict face covering measures, non-strict social isolation, and non-strict travel restrictions.

#### 3.2.2 Configuration outcomes of high morbidity

The overall consistency of the configuration for the non-high morbidity outcome was 0.787 and the overall coverage was 0.748.

##### 3.2.2.1 Configuration 1: face covering

In configuration 1, where the non-high morbidity outcome occurred, only one antecedent factor of the face covering was included. Face covering are particularly important for non-high morbidity compared to other conditions, as this factor alone constitutes a sufficient condition to explain the results. The consistency of this configuration is 0.793 and the original coverage is 0.438, indicating that this path can explain 43.8% of the cases.

##### 3.2.2.2 Configuration 2: testing and contact tracing * social isolation

In configuration 2, strict testing and contact tracing and social isolation measures are the combined configurations to achieve non-high morbidity. The consistency of this configuration was 0.862, with an original coverage of 0.525, and the path was able to explain 52.5% of the cases.

##### 3.2.2.3 Configuration 3: testing and contact tracing * travel restrictions

In configuration 3, strict testing and contact tracing measures and travel restriction measures are the combined configurations to achieve non-high morbidity. The consistency of this configuration is 0.854, the original coverage is 0.435, and this path can explain 43.5% of the cases.

### 3.3 Robustness test

Adjusting the number of case frequencies is one of the commonly used robust methods to deal with the threat of parameter setting in fsQCA (15). When performing the adequate configuration analysis, we set the case frequency number to 1, indicating that a grouping is included in the analysis as long as it covers one case. In order to avoid the impact of extreme cases on the analysis results and to ensure the robustness of the results, we adopted the method of adjusting the case frequency, and sets the minimum case frequency to 3, which means that each configuration is included in the study when it meets the condition of covering at least three cases. In this study, after we excluded configurations with a case frequency of less than 2, there are still 56 cases remaining, accounting for 81% of the total samples, meeting the requirement of retaining at least 75% cases (11). The standardized results show that the number, composition, consistency, and coverage of the configuration do not change after changing the minimum case frequency, which proves that the analysis results are reliable, as shown in Table 8.

**Table 8:** Robustness test.

Condition variables	High morbidity	Non-high morbidity		
	Configuration	Configuration 1	Configuration 2	Configuration 3
Face covering	⊗	•		
Testing and contact tracing			•	•
Social isolation	⊗		•	
Travel restrictions	⊗			•
Consistency	0.826	0.793	0.862	0.854
Original coverage	0.581	0.438	0.525	0.435
Unique coverage	0.581	0.182	0.086	0.020
Consistency of the overall solution	0.826	0.787		
Overall solution coverage	0.581	0.748		

⊗ indicates that the condition variable does not appear, • indicates that the condition variable appears, and a space indicates that the condition variable may or may not appear in the configuration.

## 4 Discussion

### 4.1 The causes of prevention and control effects are not symmetrical

According to the results of configuration analysis, the combination of non-strict face covering measures, social distancing and travel restrictions led to a high morbidity. While strict face covering can indeed be a sufficient condition for non-high morbidity in the early stages of the outbreak, non-high morbidity cases are more likely to be explained by a combination of strict testing and contact tracing and social isolation, and a combination of strict testing and contact tracing and travel restrictions. In a high-morbidity configuration, even strict testing and contact tracing may not reverse the effectiveness of outbreak control. In addition to strict testing and contact tracing, at least strict social distancing measures or strict travel restrictions are needed to be relatively effective. This is because even if the government announced strict testing and contact tracing measures at the beginning of the outbreak, on the one hand, it is difficult to achieve very strict testing and contact tracing measures due to the limited testing capacity of individual countries; on the other hand, without considering the capacity of policy implementation, the actual implementation of the policy is different. In this case, if there are no restrictions on agglomeration or travel, more families or communities may be infected.

### 4.2 Strict face covering measure is a cost-effective mean in the early stage of the epidemic

In this study, face-covering measure was found to play an important role in both the configuration that promoted high morbidity and the non-high morbidity configuration. In both the initial database of 119 cases and the final database of 69 cases, most of the countries that made it mandatory to wear masks in public places achieved better results when there were more than 100 confirmed cases. Although many of these countries have not only adopted mask-wearing measures, strict mask wearing is the consensus that these countries have achieved success in the early stages of the epidemic. Javid, a researcher from the University of Cambridge, believes that in terms of the effectiveness of limiting the spread of the virus, wearing masks can prevent asymptomatic infected people from spreading the virus, so it may affect the development track of the disease (33). This may also be one of the reasons why most of the countries that strictly required the wearing of masks at the early stage of epidemic have achieved better results.

As a basic NPIs measure, face covering has a strong protective effect for people who cannot abide by social isolation or other measures (34). It can not only prevent the virus from falling into more places from infected or asymptomatic infected people in the form of aerosols and droplets (35), but also provide protection for people who are not infected with the virus. Previous studies also confirmed that wearing a mask is effective in reducing the number of new infections while delaying the peak of fluence and significantly reducing the number of new cases (8, 36, 37). Some recent studies have also shown uncertainty as to whether wearing a mask or N95/P2 respirator helps slow the spread of respiratory viruses (38). However, there is no denying that wearing a mask is less expensive than other control measures, in the early stage of the epidemic, making it a cost-effective means of preventing and controlling the epidemic.

### 4.3 The combination of non-strict wearing of masks and containment measures resulted in a poor epidemic prevention effect

The results of the high morbidity configuration showed that non-strict social isolation or travel restrictions combined with non-strict mask were the two paths that led to poor effectiveness. Wearing masks is meant to prevent the spread of the virus through respiratory droplets, social isolation is meant to minimize the close human interactions and physical contact that can lead to the spread of deadly diseases, and travel restrictions are meant to reduce the spread of outbreaks due to the movement of people. The combination of a lack of strict mask-wearing, social isolation and travel restrictions measures means that once a person is infected, the presence of that person in public without wearing a mask can lead to more infections and spread to different areas. Even if strict testing and contact tracing measures are enacted at this time, the actual implementation is not only difficult because the spread of the epidemic has already taken shape, but also it is inevitable that there will be many asymptomatic infected persons who are difficult to track and control. Although strict containment measures may bring large economic and social costs in the short term, existing studies have proved that traffic control in various places effectively contained the spread of the epidemic, even though it may bring large costs in the short term, it also helps economic recovery as soon as possible (39).

### 4.4 Strict containment and control measures combined with active pursuit measures can achieve better prevention and control effects

The results of the non-high morbidity configuration proved that it is difficult to effectively control the spread of the epidemic with a single containment measure or testing and contact tracing measures. Argentina, for example, responded strongly to the COVID-19 pandemic by implementing strict social isolation measures to restrict assembly, such as the closure of public places and places of business, as well as severe travel restrictions on the movement of people. However, nursing homes, prisons and other detention facilities were also infected in this country, and the number of infections rose rapidly in the latter period (40). Although strict testing and contact tracing measures play an important role in achieving non-high morbidity, single testing and contact tracing measures, if not combined with certain measures of population agglomeration and movement, will bring great burden and pressure to epidemic prevention and control. Once large-scale clusters of infections occur, it is difficult to control the epidemic situation.

Strict containment measures combined with testing and contact tracing are important pathways to achieve non-high morbidity. Testing and contact tracing are important in the early stages of transmission control, through which potential asymptomatic and infectious carriers can be actively identified and promptly prevented from spreading; social isolation measures can delay the peak of the epidemic, giving health care systems a chance to be better prepared to contain it. Larger numbers of cases may overwhelm contact-tracing systems and require broader social isolation interventions, with social isolation and screening tests considered the most cost-effective alternatives to ameliorate the COVID-19 pandemic (41). The results of this study suggest that travel restrictions are as effective as social isolation as measures to limit population movement. Since it is still likely that new outbreaks will occur even after the introduction of the blockade measures. Therefore, it is necessary to take proactive measures by implementing strict detection measures, proactive case detection, and a combination of contact tracing and case isolation to avoid or minimize the impact of future outbreaks. This conclusion is also consistent with Seung’s findings that social isolation measures may not be effective without active interventions such as mass testing and contact tracing (20).

### 4.5 Timely and proactive containment strategies have better epidemic prevention

A common feature of the countries in the non-high-morbidity configuration was that relatively strict measures were taken long before the number of cases exceeded 100. This indicates that the earlier the policy stringency is improved in the early stage of epidemic prevention and control, the easier it is to achieve results. A common feature of the countries in the high-morbidity configuration was that the strictness of the policies in these countries was not improved after the number of cases exceeded 100. Even if the strictness of the policies was slightly improved in the following 6 weeks, the reality of large-scale spread of the epidemic could not be resolved. This difference may be related to the prevention and control strategies adopted in the early stage of the epidemic.

Some scholars divided the strategies of various countries into two categories. One is the mitigation strategy, in which countries do not pursue interruption of transmission, but aim to slow down the epidemic until herd immunity is established. Countries with mitigation strategies hope to avoid the collapse of their healthcare systems, as well as economic and social shutdowns. The other is the containment strategy, where the core NPIs of a containment strategy are proactive case detection and management, tracing and isolation of close contacts, and strict restrictions on the gathering or movement of people (42). These countries have generally been able to adopt stringent measures at the beginning of an outbreak or rapidly increase policy stringency to a high level, and this stringency has been sustainable.

Mitigation strategies are typically represented by Japan, United Kingdom, France, and the United States. This strategy has proved risky in the fight against COVID-19. On the one hand, the novel coronavirus has different transmission characteristics from previous influenza, that is, infected people without symptoms can also spread the coronavirus (43). On average, a patient with COVID-19 transmits the infection to two to three other people without exposure and without control measures. In the face of a highly infectious virus such as novel coronavirus, the implementation of less stringent and relatively passive mitigation strategies runs the risk of an uncontrolled outbreak. Facts have proved that although Japan, the representative country implementing this strategy, achieved good epidemic prevention and control effect in the early stage of the epidemic (the 6-week morbidity was 2.97 per 100,000). However, this strategy was unable to stop the spread of the epidemic, a second outbreak occurred in Japan in July 2020, which was more severe and had a faster increase in cases than the first wave (40). On the other hand, the reason why Japan has achieved good results in the early stage of epidemic, partly depends on the high consciousness and health literacy of the public. Other countries that chose this strategy are difficult to achieve the same effect due to their cultural concepts and other deep reasons. Countries that adopt mitigation strategies may be able to control the epidemic in the early stages; provided that timely measures can be introduced at the beginning of the epidemic and that there is a good cooperation from the public.

Containment strategies are typically represented by China, Singapore, Thailand, etc. Countries that adopted containment strategies were able to effectively control the scale of transmission even with a slight delay in the initial policies. China, an early outbreak country, had not yet reached a high level of policy stringency when the cumulative number of confirmed cases exceeded 100. However, extremely stringent and comprehensive measures were taken in a short period of time immediately after the outbreak was found to have started spreading. From February 14, 2020, the number of new daily confirmed cases dropped rapidly, and by March 7, 6 weeks after the 100 cases mark, the number of new daily confirmed cases has remained extremely low (6-week morbidity is only 5.53 per 100,000). The conclusion of this study is also similar to that of the Eubank’s study, namely, a strict and proactive containment strategy is a more feasible dealing with the epidemic (7).

## 5 Conclusion

In this study, we focus on the combination path of NPIs affecting the prevention and control effects in the early stage of the epidemic. The results showed that the causes of high morbidity are not symmetrical with the causes of non-high morbidity, strict face covering measure is a cost-effective mean in the early stage of the epidemic, and the combination of non-strict face covering and containment is the most important factor leading to poor prevention and control, and the combination of strict containment and proactive pursuit is the way to achieve superior prevention and control, timely and proactive containment strategies have better prevention and control, and should mobilize the public to cooperate.

## 6 Limitations

This study did not include factors such as national political system, cultural background, and international responsibility in the analysis. When analyzing the results and discussing them, there was no argument based on these deep-seated factors. Therefore, this study can only analyze the measure level. Therefore, the research conclusions of this study need to be tailored to the actual application of NPIs for epidemic prevention and control in various countries.

## Data availability statement

The original dataset for this study was obtained from: https://covidtracker.bsg.ox.ac.uk/. The data used for the analysis of this study have been included in the article; for further inquiries, please contact the corresponding author.

## Ethics statement

Ethical approval was not required for the study involving humans in accordance with the local legislation and institutional requirements. Written informed consent to participate in this study was not required from the participants or the participants’ legal guardians/next of kin in accordance with the national legislation and the institutional requirements.

## Author contributions

CY: Conceptualization, Data curation, Formal analysis, Methodology, Software, Writing – original draft, Writing – review & editing. QL: Data curation, Methodology, Writing – review & editing. ZZ: Conceptualization, Writing – review & editing. ZC: Conceptualization, Writing – review & editing. HG: Methodology, Writing – review & editing. DH: Conceptualization, Methodology, Writing – review & editing, Funding acquisition, Supervision. WY: Conceptualization, Methodology, Writing – review & editing, Supervision.
